# Evaluation of the frequency and predictive factors of ethanol consumption among students of the university of pharmacy of monastir

**DOI:** 10.1192/j.eurpsy.2021.1524

**Published:** 2021-08-13

**Authors:** N. Bensaid, A. Ghorbel, M. Rekik, N. Messedi, S. Hachicha, F. Messadi, J. Aloulou

**Affiliations:** 1 Toxicology Unit, hygiene Loboratory, sfax, Tunisia; 2 Psychiatry (b), Hedi Chaker University hospital, sfax, Tunisia

## Abstract

**Introduction:**

Ethanol is widely consumed by the world’s population, especially young people as part of their university life. In Tunisia, surveys and studies about consumption of ethanol among students are rare or even exceptional.

**Objectives:**

To evaluate the extent of the consumption of ethanol among pharmacy students in Monastir University, from the first year to residency, and to define the factors associated to this consumption.

**Methods:**

This study was used to collected information about the participants using a questionnaire that covers the different parameters of the consumption of ethanol during student life and the factors influencing this consumption.

**Results:**

154 participants aged 23.61 years +/- 2.63 among them 37.7% were men and 62.3% women. The prevalence of ethanol consumption was 44.8 %. The main reason for dirking was to party (79.2%). The consumption of ethanol was significantly associated with sex (p=0), place of residence (p=0.047), frequency of hang-outs (p=0), sex life (p=0) and students perception of the alcohol-health relationship (p=0). Various health problems were related to the frequency of consumption of ethanol, such as memory problems (p=0.002), violence and injuries (p=0.014).

**Conclusions:**

The findings of this study underline the need to develop specific studies and general population surveys in order to better assess the situation in Tunisia and to put in place appropriate prevention strategies, such as information and awareness campaigns, aimed at reducing or at least rationing the consumption of ethanol.

**Conflict of interest:**

No significant relationships.

